# There's No Place Like Home: Crown-of-Thorns Outbreaks in the Central Pacific Are Regionally Derived and Independent Events

**DOI:** 10.1371/journal.pone.0031159

**Published:** 2012-02-17

**Authors:** Molly A. Timmers, Christopher E. Bird, Derek J. Skillings, Peter E. Smouse, Robert J. Toonen

**Affiliations:** 1 Coral Reef Ecosystem Division, Joint Institute for Marine and Atmospheric Research, Pacific Islands Fisheries Science Center, National Oceanic and Atmospheric Administration (NOAA) Fisheries, Honolulu, Hawai'i, United States of America; 2 School of Ocean and Earth Science and Technology, Hawai'i Institute of Marine Biology, University of Hawai'i, Kāne'ohe, Hawai'i, United States of America; 3 Department of Zoology, University of Hawai'I, Honolulu, Hawai'i, United States of America; 4 Department of Ecology, Evolution, and Natural Resources, Rutgers University, New Brunswick, New Jersey, United States of America; University of Canterbury, New Zealand

## Abstract

One of the most significant biological disturbances on a tropical coral reef is a population outbreak of the fecund, corallivorous crown-of-thorns sea star, Acanthaster planci. Although the factors that trigger an initial outbreak may vary, successive outbreaks within and across regions are assumed to spread via the planktonic larvae released from a primary outbreak. This secondary outbreak hypothesis is predominantly based on the high dispersal potential of A. planci and the assertion that outbreak populations (a rogue subset of the larger population) are genetically more similar to each other than they are to low-density non-outbreak populations. Here we use molecular techniques to evaluate the spatial scale at which A. planci outbreaks can propagate via larval dispersal in the central Pacific Ocean by inferring the location and severity of gene flow restrictions from the analysis of mtDNA control region sequence (656 specimens, 17 non-outbreak and six outbreak locations, six archipelagos, and three regions). Substantial regional, archipelagic, and subarchipelagic-scale genetic structuring of A. planci populations indicate that larvae rarely realize their dispersal potential and outbreaks in the central Pacific do not spread across the expanses of open ocean. On a finer scale, genetic partitioning was detected within two of three islands with multiple sampling sites. The finest spatial structure was detected at Pearl & Hermes Atoll, between the lagoon and forereef habitats (<10 km). Despite using a genetic marker capable of revealing subtle partitioning, we found no evidence that outbreaks were a rogue genetic subset of a greater population. Overall, outbreaks that occur at similar times across population partitions are genetically independent and likely due to nutrient inputs and similar climatic and ecological conditions that conspire to fuel plankton blooms.

## Introduction

Outbreaks of the crown-of-thorns sea star, *Acanthaster planci*, are widely recognized as a major threat to coral reef ecosystems. Ecologically, outbreaks severely impact reef systems [1]. They can alter community structure [2], [3], promote algal colonization [1], [4], and affect fish population dynamics [5]–[7]. Economically, outbreaks of *A. planci* reduce the aesthetic value of coral reefs, thereby negatively impacting economies that depend on tourism. To reduce the impact of these corallivores, costly control and eradication programs have been established in several countries [8], [9]. For example, the Australian government spends about $3 million AUD per year to prevent and control outbreaks on the Great Barrier Reef (Cooperative Research Centre for the Great Barrier Reef World Heritage Area). Understanding the manner in which outbreak populations develop is critical for efficient management and conservation of coral reefs across the Indo-Pacific region.

Outbreaks may arise from a single mass recruitment event or from the progressive accumulation of sea stars from multiple cohorts [10]. Despite more than 30 years of research on crown-of-thorns outbreaks, the triggers, development, and spread of outbreaks are not fully understood. Both anthropogenic factors such as urbanization and subsequent sedimentation [11], [12], terrestrial runoff [12], [13], and overfishing [14], [15] and naturally occurring phenomena such as typhoons, hurricanes and El Niño events [13], [16], [17], heavy rainfall [13], larval retention from eddy formation [18], fluctuating current paths [19], and the transition zone chlorophyll front [20], [21] have been correlated with outbreak formation. Regardless of whether outbreaks are initially triggered from natural or anthropogenic influences, it is widely accepted that once an initial population explodes, dispersing larvae from the boom cohort will seed sequential outbreaks in a chain reaction [22]–[24]. This ‘secondary outbreak hypothesis’ was initially proposed to explain the wave of outbreaks that moved in a southerly direction along approximately 1300 km of the Great Barrier Reef (GBR) [22], [23], [25]–[28].

The foremost assumption of the secondary outbreak hypothesis is that *A. planci* larvae disperse widely, *en mass* on oceanic currents. The pan-tropical Pacific range (Australia to Panama) of *A. planci* is a potential indicator of broad dispersal, and available genetic evidence using allozyme, mitochondrial COI DNA, and nuclear microsatellites supports high dispersal ability, with few detected barriers to gene flow [26], [29]–[32]. Additionally, three testable genetic assertions underpin this hypothesis, based on the high dispersal potential attributed to *A. planci* [33]–[37], the correlated timing of secondary outbreaks in distant locations [1], [19], [23], and oceanic current patterns [20], [24], [29]. Outbreak populations are (1) genetically differentiated from non-outbreak populations, (2) genetically similar to each other, and (3) exhibit lower internal genetic diversity than do non-outbreak populations.

On the GBR, primary outbreaks exhibit a subset of the genetic diversity in the total population [25], [27], [28] and are believed to produce abnormally large cohorts of larvae that drive connectivity among disparate populations [25]–[28]. Consequently, secondary outbreaks are genetically distinct from the low-density (non-outbreak) local populations that normally inhabit reefs [25], but are not differentiated from other outbreak populations [25]–[28]. Outside of the GBR, Yasuda et al. [29] found that outbreak populations were genetically homogenous along the path of the Kuroshio current in the Ryukus Islands, but it is not known whether outbreak populations are differentiated from non-outbreak populations.

Overall, there have been very few direct tests of the secondary outbreak hypothesis. It has only been supported with genetic data in a limited portion of *A. planci's* range (along a 750 km stretch in the GBR), and is based upon dated allozyme assays. Nevertheless, this hypothesis has become an accepted theory to explain outbreaks that occur consecutively among the discontinuous coastlines of islands, archipelagos, and regions throughout the tropical Pacific Ocean [19], [20], [29], [38], [39]. Broad extrapolation beyond the GBR has resulted in the presumption that outbreaks can and do propagate across the entire range of *A. planci* [40]. For example, Houk et al. [20] propose that outbreaks triggered by the transition zone chlorophyll front in the Hawaiian Islands eventually seed secondary outbreaks over 4500 km away in the northwestern Pacific, dispersing progressively along the path of the North Pacific Gyre, although there are no data confirming dispersal over thousands of kilometers.

Here we examine the genetic structure of the highly variable mitochondrial control region (mtDNA, 530 bp) [41] of *Acanthaster planci* across the Pacific Ocean, from Yap in the western Pacific to Hawai'i and Mo'orea in the central Pacific, testing the extent to which the larvae of *A. planci* readily disperse, thereby defining boundaries to secondary outbreak propagation via larval dispersal. We specifically test the spatial scale of genetic partitioning (among sites within islands, among islands within archipelagos, among archipelagos within regions, and among regions) and the level of genetic differentiation between outbreak and non-outbreak populations. Additionally, we test for differences in the genetic diversity within and among outbreak and non-outbreak populations. The results of this study advance our understanding of the propagation of outbreaks via larval dispersal, highlight the genetic complexity of such a widespread planktonic species, and contribute to improving the efficiency and focus of current management strategies for this destructive corallivore.

## Materials and Methods

### Sample sites and collection

**Figure 1:**
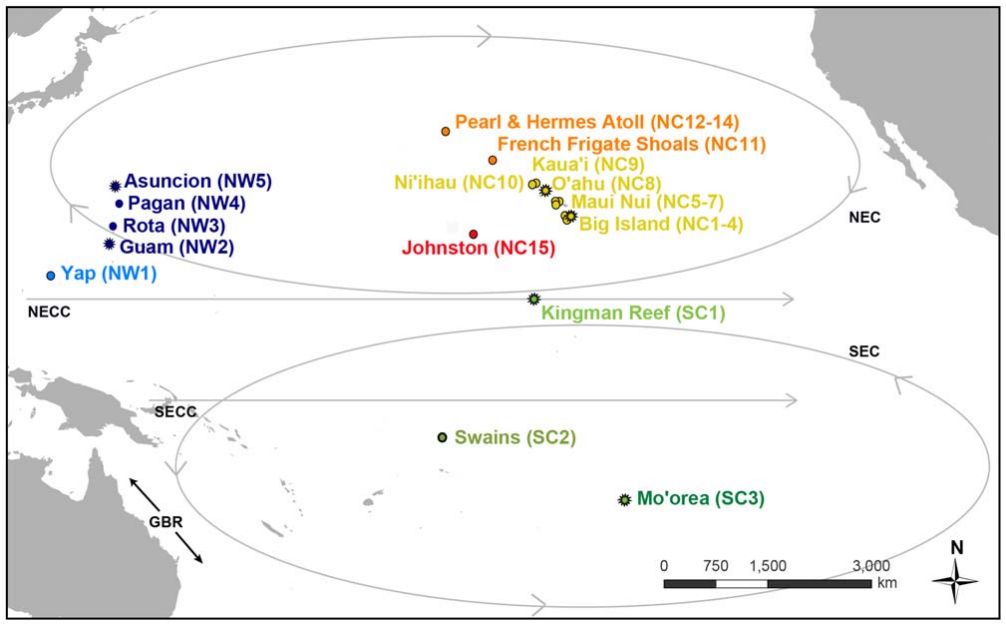
Sample locations of *Acanthaster planci* populations in the Pacific Ocean used in this study. Locations are color coded by region and shaded by subregion or archipelago. Shades of blue represent the northwestern Pacific (NW), shades of green represent the south central Pacific (SC), and red, orange, and yellow represent the north central Pacific (NC). The influential current paths in the central Pacific are represented: North Equatorial Countercurrent (NECC), North Equatorial Current (NEC), South Equatorial Countercurrent (SECC), and South Equatorial Current (SEC). GBR represents the Great Barrier Reef. Assigned location numbers that correspond to each region are represented in parentheses next to each site name and outbreak population locations are starred.

Adult *A. planci* (n = 656 sea stars) were collected between 2005 and 2008 from 23 sites across the Pacific Ocean: the northwestern Pacific (NW) region, the north central Pacific (NC) region, and the south central Pacific (SC) region (Fig. 1, Table 1). Sites classified as having outbreak populations are denoted by an * (criteria for outbreak designation are discussed below). In the NW (n = 5 sites), samples were collected from the western Caroline Islands (Yap, NW1) and four islands across a 730 km stretch in the Mariana Archipelago (Guam*, Rota, Pagan, and Asuncion*; NW2-5). In the NC (n = 15 sites), samples were collected from ten locations along 590 km of the main Hawaiian Islands (Big Island of Hawai'i: East*, South, West, and one historical population sampled in 1982; Maui Nui: Lāna'i, Maui, Moloka'i; O'ahu*; Kaua'i; Ni'ihau; NC1-10) four locations across a 1050 km stretch in the Northwestern Hawaiian Islands (Mokupāpapa/French Frigate Shoals; and Pearl & Hermes Atoll: fore reef, back reef, lagoon; NC11-14), and Johnston Atoll (NC15). In the SC (n = 3 sites), samples were collected from the Line Islands (Kingman Reef*; SC1), Samoan Islands (Swains Island; SC2), and Society Islands (Mo'orea*; SC3). In most cases, there was one location sampled per island, but note that three locations were sampled around the Big Island of Hawai'i, three locations were sampled from Maui Nui (a single island during low sea level stages and presently contiguous *A. planci* habitat for the islands of Moloka'i, Lāna'i, and Maui), and three habitat types were sampled at Pearl & Hermes Atoll (forereef, backreef, and lagoon).

**Table 1:** Summary information and statistics where outbreak occurrence is the years in which outbreaks occurred at some locations and the method by which they were detected (TD = towed diver; BT = belt transect; TS = timed swim), collection year is the year the samples were collected, N is the number of samples, H is the number of haplotypes, H_u_ is the number of unique haplotypes, h is haplotype diversity, H_e_ = 1/(1−h) is the number of effective haplotypes, and π is nucleotide diversity.

Sample Location	Outbreak Occurrence [Method]	Collection Year	*N*	H	H_u_	*h*	H_e_	π
**North Central Pacific**								
Hawai'i 1982 (NC1)		1982	44	35	15	0.981	53	0.016
Hawai'i East (NC2*)	2008 [BT]	2008	29	25	7	0.990	100	0.017
Hawai'i South (NC3)		2007	34	29	13	0.989	91	0.017
Hawai'i West (NC4)		2007	42	27	8	0.970	33	0.012
Maui Nui (Lāna'i) (NC5)		2007	30	26	10	0.984	62	0.019
Maui Nui (Maui) (NC6)		2007	26	20	8	0.982	56	0.019
Maui Nui (Moloka'i) (NC7)		2007	22	20	8	0.991	111	0.016
O'ahu (NC8*)	2005 [TD, BT]	2005	25	23	10	0.993	143	0.016
Kaua'i (NC9)		2007	24	24	7	1.000	300 ^a^	0.02
Ni'ihau (NC10)		2007	30	22	7	0.977	43	0.012
FFS (NC11)		2007	13	11	5	0.974	38	0.019
PHR (Forereef) (NC12)		2007	46	43	30	0.997	333	0.022
PHR (Backreef) (NC13)		2007	20	16	6	0.962	26	0.02
PHR (Lagoon) (NC14)		2007	58	17	4	0.828	6	0.02
Johnston Atoll (NC15)		2006	33	23	15	0.968	31	0.011
**South Central Pacific**								
Kingman Reef (SC1*)	2002-present [TD, BT]	2006	39	16	6	0.904	10	0.037
Swains (SC2)		2008	20	16	11	0.963	27	0.058
Mo'orea (SC3*)	2006–2009[ref 49]	2008	24	20	14	0.982	56	0.063
**Northwestern Pacific**								
Yap (NW1)		2007	13	11	8	0.962	26	0.019
Guam (NW2*)	2003-present [TD]	2007	27	27	20	1.000	378 ^a^	0.025
Rota (NW3)		2007	16	13	4	0.975	40	0.023
Pagan (NW4)		2007	14	10	2	0.945	18	0.018
Asuncion (NW5*)	2007 [TS]	2007	27	25	11	0.994	167	0.024

An ^a^ denotes samples with no duplicated haplotypes. PHR represents Pearl & Hermes Atoll and FFS represent French Frigate Shoals. Corresponding location numbers are in parentheses.

Live sea stars were sampled non-lethally by snipping off an arm tip *in situ*, while either free diving or scuba diving [42]. Tissue from tube feet was preserved in 95% ethanol and stored at −20°C until DNA was extracted. For the historical 1982 population, whole animals were collected and pyloric caeca were preserved in 95% ethanol before being stored at −20°C. All necessary permits were obtained for the described field studies.

### Classification and densities of outbreak populations

The density threshold at which a population of *A. planci* is considered to be an outbreak varies depending upon the survey's method and spatial scale. Due to opportunistic sampling, three different methods were used to diagnose populations as outbreaks: towed-diver surveys (a similar procedure to the established manta-tow technique), belt transects, and swim surveys (Table 1). For manta-tow surveys, where counts of sea stars are notoriously underestimated, reef areas containing >1500 non-cryptic sea stars km^−2^ (15 ha^−1^) are considered to be undergoing a population outbreak [43]. Towed-diver surveys, conducted by NOAA Fisheries' Coral Reef Ecosystem Division during Pacific Reef Assessment and Monitoring Program (RAMP) biennial research cruises to the U.S. Pacific Islands, were used to quantify the localized outbreak densities along 2 km of habitat at Guam (NW2*), Kingman (SC1*), and O'ahu (NC8*) following the manta-tow criteria [44], [45]. Densities yielded 9400 sea stars km^−2^ at NW2*, 11,050 sea stars km^−2^ at SC1*, and 50,500 sea stars km^−2^ at NC8*. For belt transect surveys, where sea stars are more thoroughly quantified, densities <10,000 km^−2^ (100 ha^−1^) are generally considered to be low density populations (non-outbreak), while values greater than this are considered to be high density populations (outbreak) [1], [8]. A single belt transect per collection site at NC8* (25 m×4 m), Hawai'i East (NC2*) (50 m×10 m), and SC1* (50 m×10 m) yielded 450,000 sea stars km^−2^ (4500 ha^−1^) [45], 350,000 sea stars km^−2^ (3500 ha^−1^), and 660,000 sea stars km^−2^ (6600 ha^−1^) respectively. In timed swimming surveys, >40 sea stars per 20 minute swim characterizes an outbreak population [46]–[48]. Divers deploying oceanographic instruments during the 2007 RAMP cruise fortuitously detected the Asuncion (NW4*) outbreak and observed >75 sea stars within a 15 minute swim. Finally the Mo'orea collections (SC3*) occurred during the outbreak event reviewed in Trapon [49].

Samples from these outbreak populations were collected between 2005 and 2008: NC8* 2005, SC1* 2006, NW5* 2007, NW2* 2007, NC2* 2008, and SC3* 2008 (Table 1). Based on RAMP towed-diver survey data, localized outbreaks at SC1* have been ongoing since 2002, and localized outbreaks around NW2* have been continuous since 2003. The NC8* outbreak has not been resurveyed since 2005 due to the inaccessibility of the site, the NW4* outbreak was not subsequently detected during the 2009 RAMP cruise, and the NC2* outbreak dispersed within two months of its detection. The SC3* outbreak was present from 2006 to 2009 [49].

### DNA extraction and PCR

Two different procedures were used for DNA extraction and amplification, based on tissue type and age of samples. DNA was extracted from tube feet, as described in Jessop [50] and Timmers [51], and DNA was extracted from pyloric caeca, using the Hotshot boiling protocol [52].

Approximately 530 base pairs of the noncoding mitochondrial DNA control region (mtDNA) were amplified with polymerase chain reaction (PCR), using the COTS-ctrl-fwd 5′-CAAAAGCTGACGGGTAAGCAA-3′ primer and the COTS-ctrl-rvs 5′-TAAGGAAGTTTGCGACCTCGAT-3′ primer [31]. For tube feet samples, 100-µL final volume PCR reactions were performed, using 30 µL of dH_2_O, 10 µL of undiluted template DNA, 10 µL of each primer (5 µM), and 50 µL of Promega MasterMix. Thermocycling was performed with an initial denaturation at 94°C for 5 min, 34 cycles (94°C for 30 s, 55°C for 1 min, 72°C for 1 min), and a final extension for 10 min at 72°C. PCR products were prepared for cycle sequencing with the UltraClean PCR Clean-Up Kit (MO BIO Laboratories, Carlsbad, CA, USA).

The PCR for historical samples utilized 25-µL reactions with 2.5 µL of 10X buffer, 5 µL of each primer (0.2 µM), 0.5 µL of undiluted template DNA, and 1.5 U of Immolase Taq polymerase (Bioline USA). Thermocycling for all samples was performed with an initial denaturation at 94°C for 5 min, 34 cycles of (94°C for 30 s, 55°C for 1 min, and 72°C for 1 min), and a final extension for 10 min at 72°C. PCR products were treated with 1.5 unit of exonuclease I and 1.5 unit of calf intestinal alkaline phosphatase (Exo-CIAP), incubated at 37°C for 60 min, and then deactivated at 85°C for 15 min.

Amplified DNA fragments were sequenced in the reverse direction and all unique and questionable sequences were repeated with an alternate sequencing primer (5′-CAATGAGAATTGCACAAGCGCCTC-3′) on an ABI 3130xl automated sequencer (Applied Biosystems Inc.). Unique haplotypes were submitted to GenBank (Accession numbers JQ397722–JQ398377).

### Data analysis

Sequences were compared and assembled using Sequencher (v4.52b; Gene Codes Corporation, Ann Arbor, MI, USA). Sequences were aligned using muscle v3.6 [53] in SeaView 4.2 [54]. Gap replacement was manually double-checked by eye using BioEdit [55].

Median-joining haplotype networks with the default weight of 10 applied to each character were created using network v4.5 (Fluxus Technology Ltd.) to illustrate haplotype variability and clustering. An analysis of molecular variance (AMOVA) was conducted using Arlequin v3.5 [56], [57] to generate a genetic distance matrix for Permanova+ [58] to run the hierarchical AMOVA and *a priori* contrast tests following Stat et al. [59]. A K2P nucleotide substitution model was determined to be the most appropriate model implemented by Arlequin for these data, as determined by Modeltest 3.7 [60]; therefore, all AMOVA analyses assumed this base substitution model. Haplotype diversity (*h*) was calculated in Arlequin and converted to the effective number of haplotypes, following Jost [61]. In the circumstance where *h* = 1 (all haplotypes are unique), the effective number of haplotypes cannot be calculated, so we calculated the effective number of haplotypes by assuming that the next haplotype sampled would be a duplicate (not unique). Nucleotide diversity (π) and population pairwise Φ_ST_ values were calculated in Arlequin. The statistical significance of pairwise comparisons were adjusted for family-wise false discovery rate, according to Benjamini et al. [62]. Effective migration rates (*N_e_m*) were calculated from pairwise *F*_ST_ values in Arlequin.

Bayesian coalescent-based calculations of within- and between-region migration rates (*N_e_m*) and mutation scaled population size (θ) were conducted using Migrate v3.2.7 [63]. One-way effective migration rates are estimated by multiplying the migration rate by the mutation scaled population size of the receiving population [63]. Four separate analyses were done: an analysis of divergence between the three major regions, with all sites within a region grouped together, and an independent analysis of each region, where each island was used as a group. For each analysis, three independent runs of a Bayesian MCMC search strategy were completed and averaged by Migrate. A nucleotide model with a transition-to-transversion ratio of 12.29∶1 was used with six regions of substitution rates and a gamma-shaped rate variation of 0.29, along with a Markov chain length = 1,000,000, sampled every 100 generations, with a 10% burn-in. Program defaults were used for all other settings. The transition-to-transversion ratio and the rate variation were calculated using Modeltest 3.7. Values for the migration rate among regions (*m*) and mutation scaled population size (θ) were taken from the highest peaks in the posterior probability distribution curves. The posterior probability distributions were examined to determine the credibility of each estimated parameter.

Pairwise Φ_ST_ values were used to assess whether genetic differentiation and geographic distance conformed to an isolation-by-distance model [64], with ordinary least squares regression in SPSS 17.0, and with the degrees of freedom adjusted to the number of samples minus one (rather than the number of pairwise comparisons minus one) in the *F* test in order to control the Type I error rate, following Bird et al. [65]. A two-way analysis of variance (ANOVA) was run to determine whether there was a difference in genetic diversity (effective number of haplotypes) between outbreak and non-outbreak populations in SPSS 17. *A priori* contrasts were performed to test for a difference in genetic diversity between outbreak and proximal low-density, non-outbreak populations. To satisfy the assumptions of the ANOVA model, the effective numbers of haplotypes were square root transformed [66].

## Results

In the sample of 656 *A. planci*, there were 341 haplotypes, of which 225 were singletons (Table 1). No haplotypes were shared across all sampling regions (NW, NC, SC). Haplotype diversity was high overall (*h* = 0.97 or 70 effective haplotypes) and ranged from *h* = 0.83–1.00 (6–333 effective haplotypes) within sampled locations (Table 1). The overall nucleotide diversity was π = 0.039, and that within sampled locations ranged between π = 0.013 and 0.041.

### Regional Comparisons

**Figure 2:**
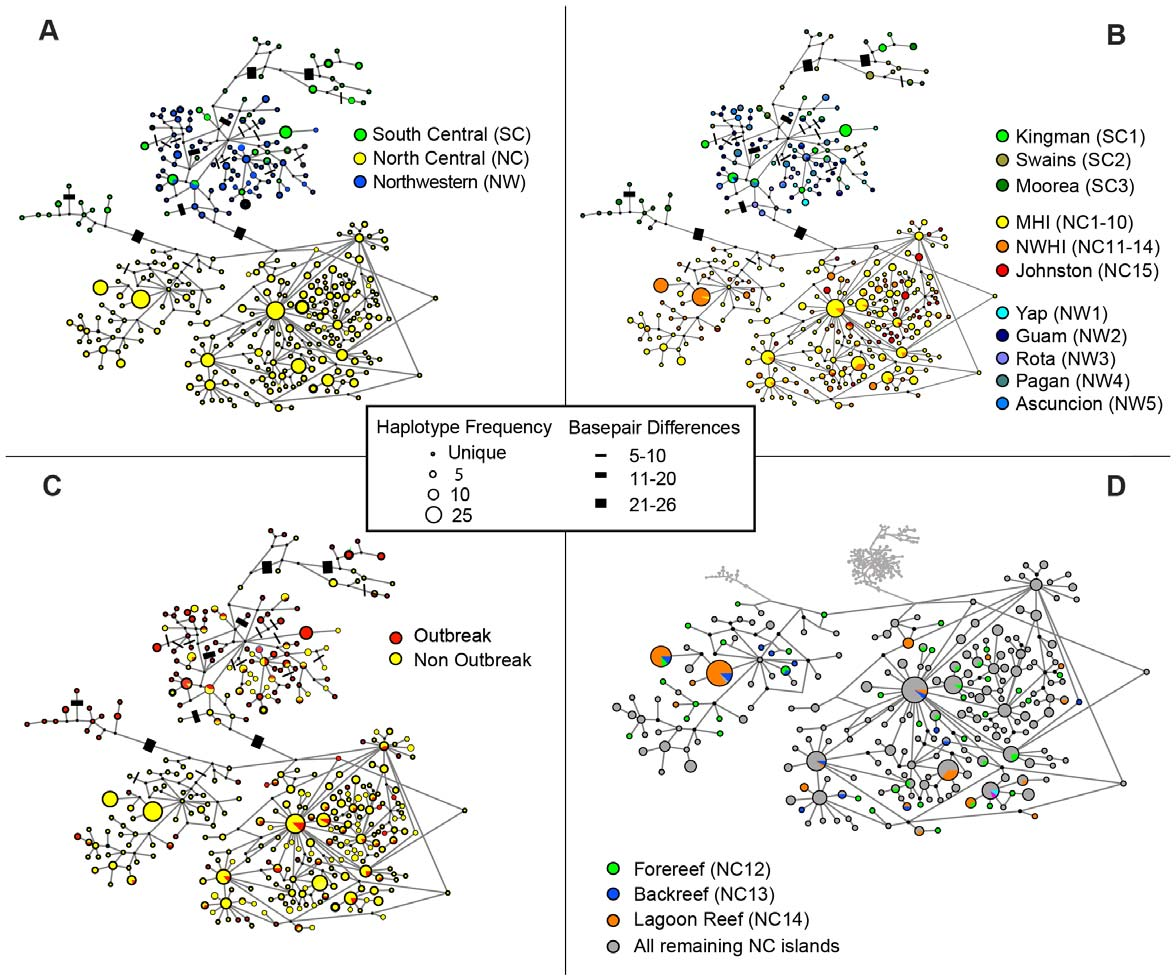
Median-joining haplotype network of *Acanthaster planci* samples. Panel A is color coded by region and panel B is color coded by island with the exception of north central Pacific (NC), which is color coded by the subregions MHI (main Hawaiian Islands) and NWHI (Northwestern Hawaiian Islands). Panel C is color coded by outbreak and non-outbreak, and panel D is coded by habitat at Pearl & Hermes Atoll. Corresponding location numbers are in parenthesis. Each circle represents a unique haplotype connected by a line to those that differ by one or more base pairs. Those lines that represent ≥5 bp differences were labeled by barred increments; however, lines are not drawn to scale. Nodes on the lines indicate missing haplotypes. The smallest colored circles represent a singleton haplotype, and the largest circle represents 25 individuals.

**Figure 3:**
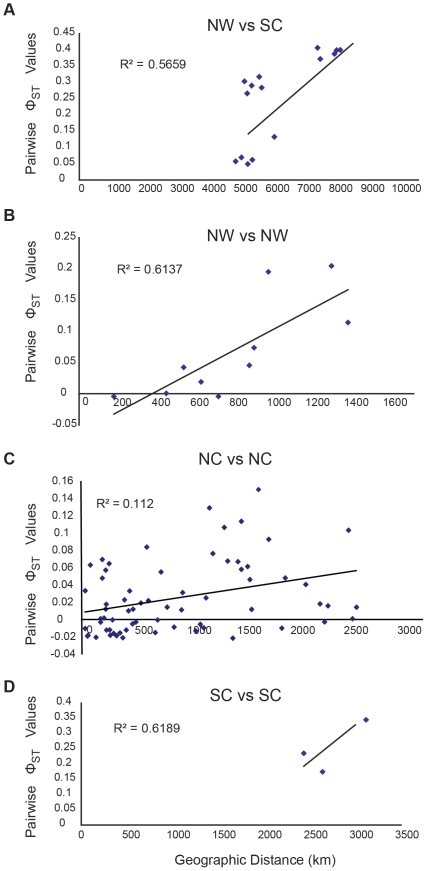
Relationships between genetic and geographical distance for *Acanthaster planci*. Patterns of isolation-by-distance: (A) across northwestern Pacific (NW) and south central Pacific (SC), (B) within NW, (C) within SC and (D) the main Hawaiian Islands (MHI) and the Northwestern Hawaiian Islands (NWHI) within the north central Pacific (NC).

**Figure 4:**
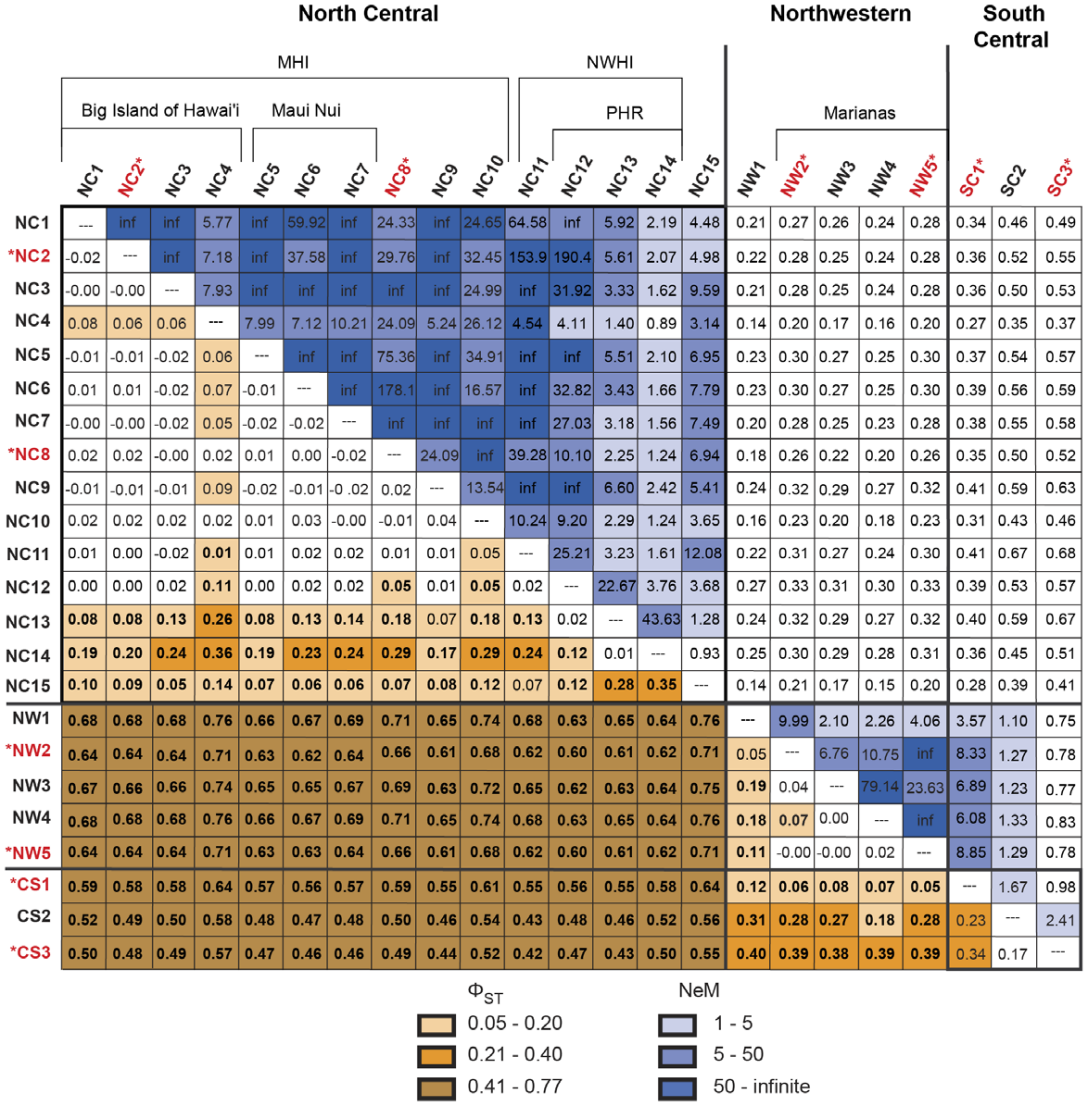
Pairwise Φ_ST_ (below diagonal) and effective migration rates (above diagonal) for *Acanthaster planci* population samples within each Pacific region. Statistically significant, accounting for family-wise false discovery error rate, is noted in bold face type (α* = 0.0354). PHR is Pearl & Hermes Atoll, MHI is main Hawaiian Islands, and NWHI is Northwestern Hawaiian Islands. Island codes are as follows: NC1 Hawai'i 1982, NC2* East Hawai'i, NC3 South Hawai'i, NC4 West Hawai'i, NC5 Lāna'i, NC6 Maui, NC7 Moloka'i, NC8* O'ahu, NC9 Kauai'i, NC10 Ni'ihau, NC11 French Frigate Shoals, NC12 PHR forereef, NC13 PHR backreef, NC14 PHR lagoon, NC15 Johnston Atoll, NW1 Yap, NW2* Guam, NW3 Rota, NW4 Pagan, NW5* Asuncion, CS1* Kingman Reef, CS2 Swains Island, and CS3* Mo'orea. Outbreak populations are * and in red.

**Figure 5:**
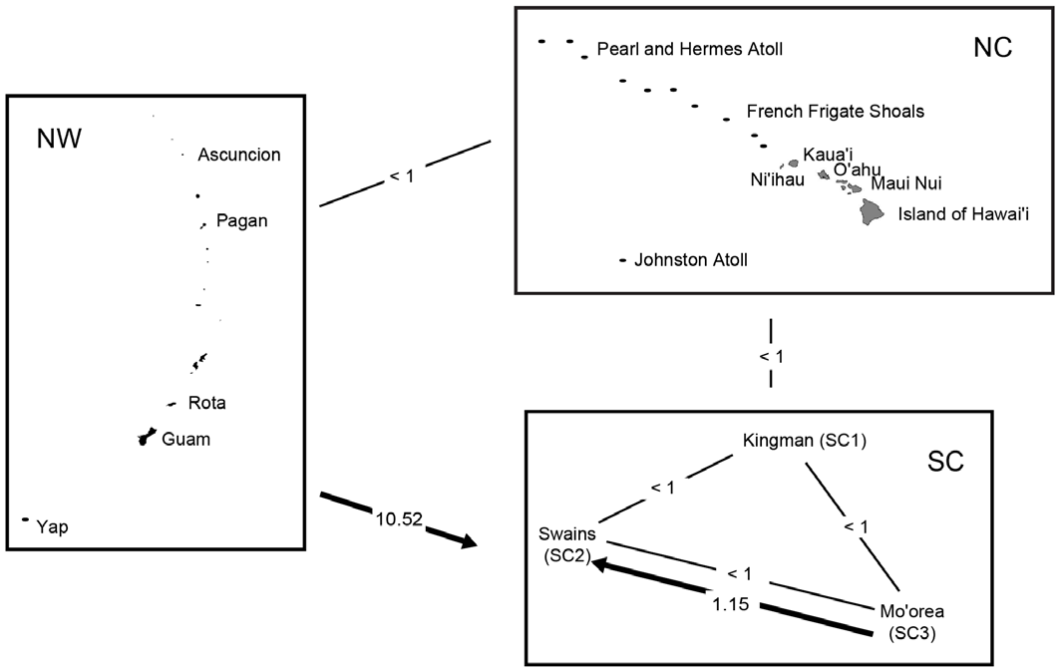
MIGRATE analysis of source and sink dispersal pathways of *Acanthaster planci* populations between regions in the central Pacific. The arrow points to the recipient region. Where arrows do not exist, *N_e_m* estimates were <1 effective migrant per generation. Region codes are as follows: SC = south central Pacific, NC = north central Pacific, and NW = northwestern Pacific.

A median-joining network reveals a strong association between haplotype identity and geographic location. Haplotypes clustered regionally, with the north central Pacific (NC; Hawaiian Archipelago, and Johnston Atoll) being completely different from those of the south central Pacific (SC; Kingman, Swains, and Mo'orea) and northwestern Pacific (NW; Yap and Mariana Archipelago; Fig. 2a, Fig. S1). Between the SC and NW, only five out of 138 haplotypes are shared, specifically between Kingman Reef (SC1*) and all sites in the NW. Regional partitioning among the NC, NW, and SC is confirmed by AMOVA (Φ_CT_ = 0.60, *P*<0.001; Table 2). Orthogonal *a priori* contrast tests demonstrated that the NC is substantially differentiated from the SC and NW (Φ_1_ = 0.61, *P*<0.001; Table 2) and that the SC is less differentiated from the NW (Φ_2_ = 0.17, *P*<0.001). Fifty seven percent of the variation in pairwise Φ_ST_ between samples in the NW and SC could be explained by the distance between the sites (*F*_1,6_ = 16.95, *P* = 0.006; Fig. 3a). SC1* is substantially more similar to the NW samples (0.05≤*Φ*_ST_≤0.13) than to either Swains (SC2) (0.27≤*Φ*_ST_≤0.32) or Mo'orea (SC3*) (0.37≤*Φ*_ST_≤0.41; Fig. 4). Migrate inter-regional analyses provided a clear unimodal peak and effective migration rates were uniformly low (*N_e_m*<1), with the sole exception of the one-way migration from the NW into the SC region (*N_e_m* = 10.52, Table 3, Fig. 5).

**Table 2:** Nested AMOVA for *Acanthaster planci* population samples, where region and sampling location nested within region are the two hierarchical factors.

Source of Variation (contrast tests indented)	df	MS	Var Comp	Notation	Φ	*P*-value
**Region**	2	1507.5	10.33	Φ_CT_	0.60	<0.001
NC vs SC & NW	1	2822.4	10.64	Φ_1_	0.61	<0.001
SC vs NW	1	192.7	1.40	Φ_2_	0.17	<0.001
**Population Samples (Region)**	20	31.4	0.89	Φ_SC_	**0.13**	**<0.001**
*Between Subregions*						
NW1 vs NW2-5	1	23.6	0.77	Φ_3_	**0.11**	**0.001**
NC1-10 vs NC11-14	1	86.8	0.37	Φ_4_	**0.06**	**<0.001**
*Outbreaks vs Non-outbreaks within Archipelagos*						
NC1-10	1	2.9	−0.02	Φ_5_	−0.00	0.733
NW2-5	1	16.0	0.25	Φ_6_	**0.04**	**0.013**
*Outbreaks vs Non-outbreaks within Region*						
SC	1	74.9	1.94	Φ_7_	**0.24**	**0.001**
*Between Outbreaks within Archipelagos*						
NC2* vs NC8*	1	5.6	0.05	Φ_8_	0.01	0.207
NW2* vs NW5*	1	6.0	−0.03	Φ_9_	−0.01	0.498
*Between Outbreaks within SC region*						
SC1* vs SC3*	1	216.1	6.84	Φ_10_	**0.53**	**<0.001**
*Between Islands with Multiple Samples in NC*						
NC12-14 vs NC5-7 & NC1-4	1	125.2	0.74	Φ_11_	**0.11**	**<0.001**
NC5-7 vs NC1-4	1	6.4	0.02	Φ_12_	0.00	0.167
*Between Years on the Big Island of Hawai'i*						
NC1 vs NC2-4 (1982 vs 2000's)	1	7.6	0.05	Φ_13_	0.01	0.087
*Between Habitats within Pearl & Hermes Atoll*						
NC12 vs NC13-14 (forereef vs other hab.)	1	39.5	0.58	Φ_14_	**0.09**	**0.001**
NC13 vs NC14 (lagoon vs backreef)	1	8.2	0.09	Φ_15_	0.01	0.191
**Within Population Sample Error**	636	6.001	6.0010			
**Total**	658					

While it is common to present an ANOVA table with pre-planned contrast tests, it is uncommon for AMOVA. To provide a bearing, we explicitly label the common tests of region and samples nested within region as Φ_CT_ and Φ_SC_ [50]. AMOVA estimates of genetic differentiation/fixation for each contrast are labeled sequentially Φ_1–13_. These thirteen *a priori* linear contrasts were performed to test the genetic differentiation among regions (NC = north central, NW = northwestern, SC = south central), between outbreak and non-outbreak population samples nested within regions, between pairs of outbreak populations nested within their respective regions, between the Mariana Archipelago and Yap in the western Caroline Islands (NW), between the main Hawaiian Islands (MHI; NC1-10) and the Northwestern Hawaiian Islands (NWHI; NC11-14), among the highly sampled island populations at Pearl & Hermes Atoll (PHR; NC12-14), Maui Nui (Maui, Lāna'i, and Moloka'I; NC5-7), and the Big Island of Hawai'I (NC1-4), among habitats sampled within PHR, and between the samples from 1982 (NC1) and the 2000s on the Big Island of Hawai'I (NC2-4). An * denotes an outbreak population. Statistically significant Φ values are listed in bold face type.

**Table 3:** Estimated migration rates calculated in MIGRATE where the estimates of migration are separated by direction; columns are source populations and rows are recipient populations.

Regions			
	NC	SC	NW
NC	-	0.04	0.04
SC	0.02	-	10.52
NW	0.02	0.83	-

SC Region			
	SC1*	SC2	SC3*
SC1*	-	0.00	0.02
SC2	0.02	-	0.02
SC3*	0.00	1.18	-

The value of *M* calculated by MIGRATE was multiplied by θ, as calculated by MIGRATE, of the destination population to estimate migration. The upper table displays pairwise migrations rates between major regions. The lower table displays pairwise migration rates between islands within the SC region. All other within-area migration rates could not be calculated with precision and so are not displayed. Abbreviations are as follows: NC = north central Pacific, SC = south central Pacific, and NW = northwestern Pacific, SC1* = Kingman Reef, SC2 = Swains Island, SC3* = Mo'orea. An * denotes an outbreak population.

### Subregional Comparisons

There is significant partitioning among sites sampled within the NW, NC, and SC regions (Φ_SC_ = 0.13, *P*<0.001; Table 2), and median-joining network reveals an association between haplotype and subregion, with most haplotypes being restricted to a single subregion (Fig. 2b, Fig. S2). There is significant partitioning of *A. planci* populations between the two sampled archipelagos in the NW region (Φ_3_ = 0.11, *P*<0.001; Table 1), among the three sampled archipelagos in the SC region (0.17≤Φ_ST_≤0.34, *P*<0.001; Fig. 4), and between the main and Northwestern Hawaiian Islands in the NC region (Φ_4_ = 0.06, *P*<0.001; Table 2, Fig. 2b, Fig. S2). Overall, 62% of the genetic variation within the NW region is explained by geographic distance (IBD) among sampling locations (*F*_1,3_ = 12.70, *P* = 0.038; Fig. 3b). In the NC, however, only 11.2% of the variation in Φ_ST_ is explained by geographic distance (*F*_1,10_ = 8.08, *P* = 0.013; Fig. 3c).

Taking the available data, there is significant population divergence among the archipelagos sampled in the SC region (0.17≤Φ_ST_≤0.34, *P*<0.001; Fig. 4). These data suggest that 62% of the variation in differentiation among these islands could be explained by geographic distance, but the result is not statistically compelling, given only three data points (*F*_1,1_ = 1.62, *P* = 0.424; Figs. 2b, 3d, S2).

Intra-regional estimates of *N_e_m* from Migrate could only be obtained among SC sites (Table 3, Fig. 5). Within the SC, there is less than one effective migrant per generation in all pair-wise comparisons, with the exception of the one-way migration from SC3* to SC2 (*N_e_m* = 1.18; Table 3; Fig. 5). The separate analyses within NW and NC did not return posterior probability distributions with single unimodal peaks; most of the posterior probability distributions for the within regional analyses were flat and inestimable. Individually estimated *N*_e_ and *m* values derived from each Migrate analysis can be found in Table S1 and Table S2.

### Relationship between outbreak and non-outbreak populations

**Figure 6:**
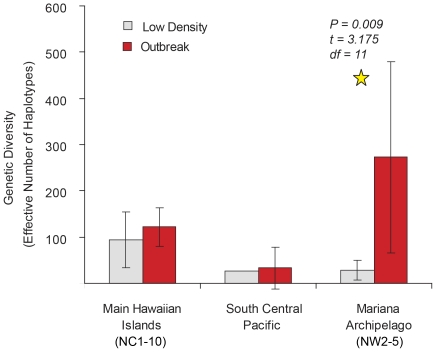
Genetic diversity measurements based on effective haplotypes between outbreak and non-outbreak populations of *Acanthaster planci* within three regions.

We found little evidence for outbreak populations being a rogue subset of the background population. The median-joining haplotype network is not consistent with the hypothesis that outbreaks are distinct from non-outbreak populations (Fig. 2c, Fig. S3). In a two factor ANOVA, there is no significant difference in haplotype diversity between outbreak and non-outbreak populations overall (*F* = 4.56, *P* = 0.06; Fig. 6). The only instance of a difference in diversity between outbreak and non-outbreak populations was in the NW region, where, contrary to the secondary outbreak prediction, outbreak populations exhibited substantially greater haplotype diversity (*H*_e_ = 273±149) than did non-outbreak populations (*H*_e_ = 29±16), with (*t*_11_ = 3.18, *P* = 0.01; Fig. 6).

Outbreak (NC2*, NC8*) and non-outbreak populations in the main Hawaiian Islands (MHI, NC region) are not significantly differentiated (Φ_5_<−0.01, *P* = 0.73; Table 2, Fig. 4). In *post hoc* pairwise comparisons, the outbreaks were not significantly different from any of the other MHI populations, with the exception of NC4 (Fig. 4), which is differentiated from the majority of MHI samples. These results suggest little impediment to larval exchange among most MHI sample locations.

In the Mariana Archipelago (NW region), outbreak (NW2*, NW5*) and non-outbreak populations (NW3, NW4) are significantly differentiated (Φ_6_ = 0.04, *P* = 0.01; Table 2). Pairwise comparisons (Fig. 4) indicate a complex pattern, however, where the NW5* outbreak is not significantly different from non-outbreak populations NW3 (Φ_ST_ = 0.02, *P* = 0.16) and NW4 (Φ_ST_ = 0.00, *P* = 0.42) and the NW2* outbreak is not significantly different from non-outbreak NW4 (Φ_ST_ = 0.04, *P* = 0.05).

In the SC, outbreak SC1*, SC3* and non-outbreak SC2 populations are also different (Φ_7_ = 0.24, *P*<0.001; Table 2), but our sampling was comparatively sparse in this region, so we can only distinguish inter-archipelagic differences.

### Relationship between outbreak populations

Outbreak populations are significantly differentiated between archipelagos (0.05≤Φ_ST_≤0.65, *P*≤0.001; Fig. 4), but they are not divergent within archipelagos. The MHI outbreak populations (NC2*, NC8*) are genetically similar (Φ_8_ = 0.01, *P* = 0.22; Table 2) and the effective migration rate between these two populations is high (Fig. 4). Likewise, the Mariana Archipelago outbreak populations (NW2*, NW5*) are not differentiated (Φ_9_<−0.01, *P* = 0.50).

### Within-subregion and within-island fine-scale spatial analyses

**Figure 7:**
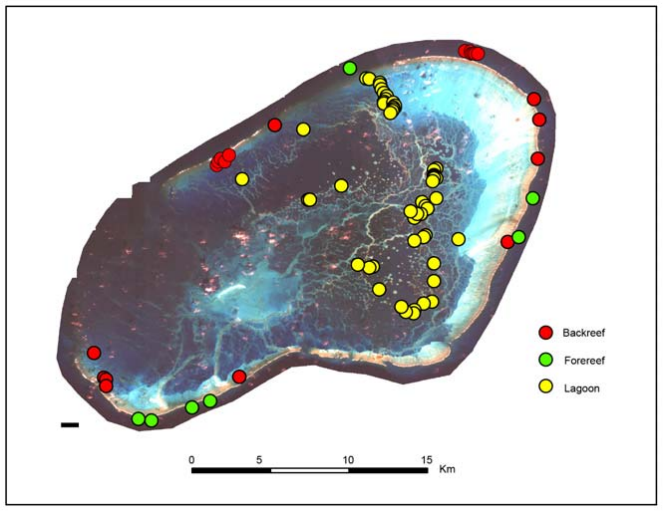
Geographic location of *Acanthaster planci* samples collected at Pearl & Hermes Atoll.

We sampled *A. planci* populations at fine scale from three specific locations in the Hawaiian Archipelago: 1) in the NWHI at Pearl & Hermes Atoll (PHR, NC12-14; Fig. 7), and in the MHI at 2) the Maui Nui complex (NC5-7), and 3) around the Big Island of Hawai'i (NC1-4). Given the high genetic diversity of control region haplotypes, we wanted to see whether increased sample size would increase our ability to detect genetic differentiation using pre-planned contrasts (Table 2). PHR was found to be significantly differentiated from both Maui Nui and the Big Island of Hawai'i (Φ_11_ = 0.11, *P*<0.001), but Maui Nui and the Big Island are not convincingly differentiated (Φ_12_ = 0.00, *P*>0.16; Table 2).

Around the Big Island of Hawai'i, there was no detectable genetic difference between the 1982 collection (NC1) and the more recent collections (NC2-4; Φ_13_ = 0.01, *P* = 0.09). In contrast, pairwise analysis revealed differences between Hawai'i West (NC4) and all three other Big Island sites (0.06≤Φ_ST_≤0.08, *P*≤0.002), though NC1-3 appeared to be panmictic (Φ_ST_≤0.00, *P*>0.38). Within the Maui Nui complex, pairwise analysis also revealed genetic panmixia (Φ_ST_≤−0.02, *P*>0.65; *N_e_m* = ∞).

**Figure 8:**
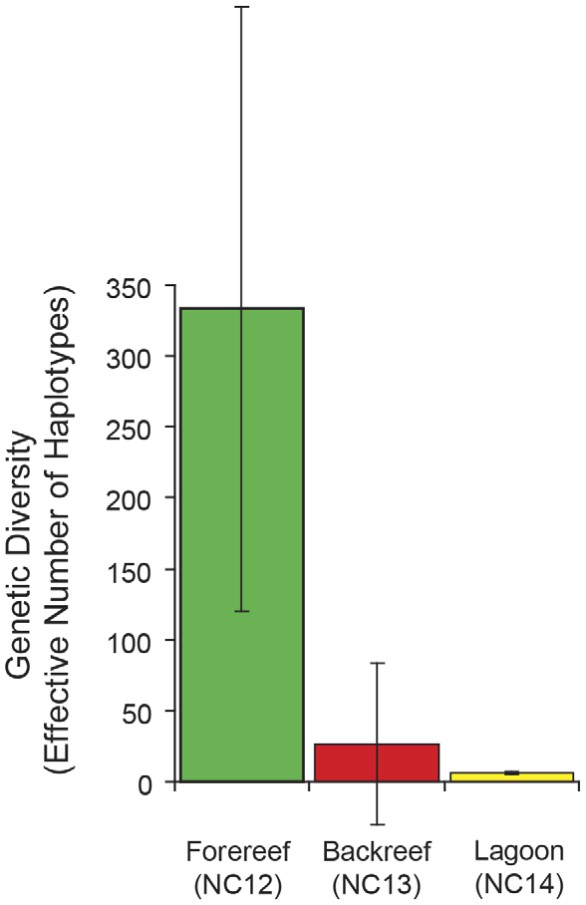
Genetic diversity measurements of *Acanthaster planci* based on effective haplotypes between habitats at Pearl & Hermes Atoll.

At a very fine spatial scale within PHR (Fig. 7), sea stars on the forereef (NC12) were significantly differentiated from those in the lagoon (NC13) and backreef (NC14), (Φ_14_ = 0.09, *P*<0.001; Table 2), but the backreef and lagoon were not significantly differentiated from one another (Φ_15_ = 0.01, *P* = 0.17). Figure 2d and Figure S4 highlights the haplotypes from these three habitats and their positions within the median-joining network. Pairwise analysis revealed meaningful differences between lagoon and forereef habitats (Φ_ST_ = 0.12, *P* = 0.001) but a trivial difference between backreef and forereef habitats (Φ_ST_ = 0.02, *P* = 0.14). The lagoon population showed divergence from all MHI populations (0.17≤Φ_ST_≤0.36, *P*<0.001). Similarly, the backreef population was divergent from all MHI populations (0.08≤Φ_ST_≤0.28, *P*≤0.03), except NC9 (Φ_ST_ = 0.07, *P* = 0.05). However, the forereef population was genetically similar to all MHI populations (0.00≤Φ_ST_≤0.02, *P*≥0.12), with the exception of NC4, NC8*, and NC10, (0.05≤Φ_ST_≤0.11, *P*≤0.025). Sea stars on the forereef exhibited substantially greater genetic diversity than on the backreef and lagoon areas of PHR (forereef *H*_e_ = 333, backreef *H*_e_ = 26, lagoon *H*_e_ = 6; Table 1, Fig. 8). The lagoon exhibited a strict subset of the diversity on the forereef, with the lowest number of effective haplotypes for any of the populations sampled (Table 1).

## Discussion

Our data show that *Acanthaster planci* populations are much more finely structured than previously hypothesized, with population structure among regions in the central Pacific, among archipelagos within regions, among some islands within archipelagos, and even among some sites around the same island. Standard theory [67] suggests that at equilibrium, the degree of population structure, *F*_ST_ (*Φ*_ST_, for haploid mtDNA markers), represents a balance between local effective size (*N*_e_) and the per generation migration rate (*m*), at least where the migration rate (*m*) is orders of magnitude higher than the recurrent mutation rate (*μ*). In keeping with that expectation, Nishida and Lucas [30] reported high levels of gene flow and low genetic structure, inferred from slowly mutating allozyme markers, among *A. planci* populations in the Pacific (though they did identify restricted gene flow between Hawai'i and other locations). On the other hand, hypervariable markers, for which *μ*>*m*, typically show finer-scale structure (more divergence among populations) because population divergence is enhanced by mutations that accumulate locally, much faster than they can be spread among populations by migration. In keeping with that expectation, Yasuda et al. [29], using nuclear microsatellite loci, reported that some groups of islands were genetically distinct. The highly mutable D-loop haplotypes from mitochondria yield outcomes more in keeping with Yasuda's results than they do with the earlier allozyme results. Of course, the secondary outbreak hypothesis was never intended to be an equilibrium prediction, but the absence of shared haplotypes between regions (Fig. 2) clearly indicates that there is virtually no long-distance exchange among archipelagos. Gene flow restrictions inferred with mtDNA sequence and microsatellites have been identified at similar spatial scales in several disparate marine species with high dispersal potential [68]–[72], suggesting that the level of partitioning exhibited here with *A. planci* is a fairly common finding.

### Outbreak propagation at large geographic scales via larval dispersal

Given the level of population structure and restriction to gene flow (Fig. 2a, Table 2), population outbreaks of *A. planci* are not spreading among regions in the central Pacific via larval dispersal. Local variation is locally derived, rather than being a consequence of long-distance immigration (Table 2, Fig. 2a,b,c; Fig. S1, S2, S3). For example, Hawaiian *A. planci* are completely differentiated from all other samples and comprise an isolated haplogroup that is 21–26 bp divergent from those in the NW and SC, representing at least thousands of years of complete isolation. Hence, *A. planci* outbreaks in the Hawaiian Islands do not spread to the NW region, as proposed by Houk et al. [20]. The timing of chlorophyll-*a* accumulation, spawning of *A. planci* in the Hawai'i region, and the subsequent outbreaks in the Mariana Archipelago was coincidental. The transition zone chlorophyll front may have triggered the outbreaks in the Hawaiian Islands, but the outbreaks that appeared in the Mariana Archipelago were independently derived. In agreement with our results, Nishida and Lucas [30] described the Hawaiian population as most differentiated from other Pacific populations, based on allozyme analyses. In contrast, using COI mtDNA, Vogler et al. [32] found *A. planci* to be panmictic in the Pacific Ocean forming a single species complex. However, the marker used by Vogler et al. [32] is known to have a lower mutation rate than that used here (control region mtDNA) [73].

Our data also do not support the possibility of outbreaks spreading via larval dispersal from SC to the NW, via the North Equatorial Current, or from the NW to the SC, via the North Equatorial Countercurrent. The few shared haplotypes between these two regions suggest the occasional exchange of larvae, possibly through Kingman Reef (SC1*), as evidenced by lesser genetic differentiation (Fig. 3a), though the shared haplotypes could also reflect ancestral polymorphism or ancient gene flow, rather than present-day connectivity [74]–[77].

Likewise, it is improbable that the outbreak populations sampled here, within archipelagos of the SC region, are spreading via larval dispersal on the South Equatorial Countercurrent or the Southern Equatorial Current and gyre. These populations reflect an isolation-by-distance pattern, with little evidence for a distinct outbreak population spreading across the region (Fig. 3c). Similarly, Yasuda et al. [29] found genetic isolation with microsatellites between Mo'orea (SC3*) and Fiji outbreak populations. Both sets of results suggest that archipelagos within this region are effectively isolated, that connectivity between archipelagos is of very low magnitude over management-relevant time-scales, and that outbreaks are not spreading via larval dispersal.

### Outbreak propagation via larval dispersal within archipelagos

#### Main Hawaiian Islands

The majority of *Acanthaster planci* samples appear to represent a single population across the 590 km stretch of the MHI, thus outbreaks may spread via larval dispersal here. High density outbreak populations of *A. planci* exhibited comparable haplotypic diversity and were genetically indistinguishable from low density populations, with the exception of the West Hawai'i sample (NC4). As in vermetid snails (*Dendropoma gregaria*, *D. platypus*, and *D. rhyssoconcha*) [69] and yellow tang (*Zebrasoma flavescens*) [70], the low density NC4 population of *A. planci* was slightly differentiated from the other MHI samples, hinting that finer-scaled population partitioning may occur. It is difficult to predict whether and exactly how an outbreak might propagate through the archipelago. The prevailing ocean currents that run along the 590 km stretch of the MHI are haphazard, due to eddys, mesoscale instability, and seasonal variability [78], [79], which could result in erratic patterns of larval exchange.

#### Mariana Archipelago

Support for the secondary outbreak hypothesis along a 730 km stretch of the Mariana Archipelago is somewhat equivocal. Following the predictions of this hypothesis, the Mariana outbreak populations are (1) genetically similar to one another and (2) genetically dissimilar to the non-outbreak populations (Table 2; Fig. 4). Contrary to its predictions, however, the outbreak populations exhibit greater haplotype diversity than do non-outbreak populations (Fig. 6; Table 1). We would expect outbreak populations to exhibit lower haplotype diversity than a non-outbreak population if outbreak populations were comprised of a rogue subset of the available array of haplotypes. Likewise, a strong IBD relationship suggests spatial isolation of the islands and larval dispersal restrictions. Furthermore, the NW5* outbreak was not significantly differentiated from non-outbreak populations NW3 and NW4, and the NW2* outbreak was not significantly differentiated from non-outbreak NW4. Sample site density was coarser in the Mariana Archipelago than in the Hawaiian Archipelago, and expanded sampling within the Mariana Archipelago should be conducted to better understand whether outbreaks spread via mass dispersal events or are strictly localized events for single islands.

### Outbreak propagation via larval dispersal within islands

Barriers to gene flow across small channels between islands and groups of islands have been identified in a number of high dispersal species [65], [68]. Intra-island barriers and gene flow restriction among habitats within atolls are less common (but also see Faucci [69], Eble et al. [70], and Barshis et al. [71]). The West sample (NC4) from the Big Island of Hawai'i is differentiated from the East and South samples (NC2*, NC3; Fig. 4) [31], suggesting that secondary outbreak propagation is restricted to certain regions within this island. A gene flow restriction along the west side has been detected in other marine species [69], [70] and could be a result of anticyclonic eddies and submesoscale circulation around the Big Island of Hawai'i [31], [69], [70], [80].

Despite the high potential for pelagic larval dispersal in this species, we detected a substantial level of differentiation between forereef and lagoon populations at Pearl & Hermes Atoll (<10 km, Fig. 7). For species with long-lived planktotrophic pelagic larvae (14 days, minimum for *A. planci* [34]), this is among the finest geographic scale of population partitioning of which we are aware [81], [82]. The drastic reduction of haplotypic diversity in the lagoon, relative to the forereef, in a species with the dispersal potential of *Acanthaster*, suggests that the lagoonal population has not reached equilibrium (Fig. 8). It is unlikely that the present pattern represents a founder effect, following submergence due to sea level rise ∼6000 years ago and restricted gene flow since, as *Acanthaster* colonized the lagoon. Rather, this pattern may either be indicative of increased larval retention and sweepstakes recruitment [83]–[85] or natural selection and specialization of sea stars within the warmer, shallow (<3 m) lagoon, relative to the cooler, deeper forereef waters (sensu [71], [72]). In either case, the observed pattern suggests that *Acanthaster* is not realizing its full dispersal potential within the restricted spatial scale of a single atoll, and if an outbreak were triggered in either habitat, it will probably not spread to the other via larval dispersal.

### Population connectivity and outbreak populations

The hypothesis that outbreaks are the primary source of connectivity for this species is based on previous findings along a 750 km stretch of the Great Barrier Reef, where low-density populations exhibited greater isolation-by-distance slopes and higher pairwise *F*_ST_ values than do the high-density populations [25], [40]. With few discernable genetic differences between high- and low-density populations, either within or between the central Pacific regions investigated here (Fig. 1), our data are not consistent with earlier studies. In the majority of cases here, outbreaks are comprised of many local genotypes, rather than being concentrated within restricted maternal lineages. Thus, it is unclear whether or not outbreaks drive genetic connectivity patterns, because increased population density could conceivably increase larval production and the number of migrants, or could increase the amount of polyspermy (fertilization of an egg by multiple sperm) and inviable larvae [86]. Though we cannot conclusively test whether outbreaks drive connectivity among islands, we did find limited connectivity among regions and archipelagos and greater connectivity within archipelagos. Hence, the data strongly suggest that larvae from neither high nor low density *A. planci* populations are, *en mass*, crossing large expanses of open ocean between archipelagos.

The geologic differences between the continental nature of the GBR and the broadly separated volcanic island archipelagos in the central Pacific could be one of the reasons behind the contrasting patterns of connectivity among *A. planci* populations within these domains. The GBR is on the comparatively shallow Australian continental shelf along a large and fairly contiguous coastline with more than 2900 reefs and 900 islands all of which provide suitable *A. planci* habitat. The current patterns along this relatively linear coastline facilitate larval dispersal up and down the coast which may support secondary outbreaks [18], [24]. The oceanic islands of the central Pacific, on the other hand, rise from the ocean floor with no continental shelf or coast to direct currents. Few oceanic islands are connected by contiguous crown-of-thorns habitat and thus larvae are less likely to immediately find suitable settlement substrate unless they are retained within their natal reef. This may explain why dispersal appears to be haphazard in the central Pacific and why, unlike the GBR, secondary outbreaks are improbable across Pacific Archipelagos.

### The Cause of Outbreaks

Given the degree of genetic portioning in *A. planci*, synchronized sequential outbreaks in disparate archipelagos are probably driven by similar environmental conditions. Periodic boom-and-bust cycles are extremely common among echinoderm populations and are typically the product of environmental and anthropogenic events that enhance phytoplankton food biomass for larvae, rather than being a reflection of a dispersal phenomenon [87]. An enhanced level of nutrients from both natural [13], [20], [21] and anthropogenic [11], [12] sources has been proposed as a major cause for *A. planci* outbreaks.

Birkeland [13] correlated outbreak prevalence among high islands across the central and western Pacific with heavy rainfall and typhoon induced terrestrial runoff. He hypothesized that the heightened nutrients in the water column from these large-scale storm events triggered phytoplankton blooms that independently increased *A. planci* larval survivorship, settlement, and *A. planci* densities around high islands. This ‘terrestrial run-off hypothesis’ was further supported by the rare occurrence of documented outbreaks on nearby nutrient poor atoll and low island systems. Similarly, Fabricius et al. [12] argued that the onset of outbreaks on the GBR is predominantly controlled by phytoplankton availability, which is governed by flooding rivers and elevated nutrient inputs. With the exception of Kingman Reef, outbreak locations in this study were found at high islands and of the high islands, the outbreaks were generally in the vicinity of rivers and watersheds (with the exception of Mo'orea and Asuncion). If higher nutrient loads do drive outbreaks [11]–[13], [20], [21], then mitigating land-based sources of nutrients would be a more effective management strategy than physically eradicating this corallivore, with the hope of precluding outbreak propagation in distant archipelagos.

At smaller spatial scales, it is not clear whether successive outbreaks in the central Pacific are a reflection of mass dispersal events or coincidentally arise from similar environmental or anthropogenic factors. Fine intra-island structure (i.e., the forereef and lagoon at PHR and West Hawai'i versus the other Big Island of Hawai'i sites) and the genetic similarity between outbreak and non-outbreak populations found within archipelagos is inconsistent with the secondary outbreak hypothesis. However, the lack of divergence between outbreak and non-outbreak populations within archipelagos indicates that populations are exchanging gametes via dispersal.

To be conservative, one should assume that outbreaks might spread between locations that do not exhibit genetic population structure, while realizing that a lack of structure does not prove evidence that outbreaks are spreading in this fashion. Our recommendation to managers is to consider seriously the role that environmental conditions and local nutrient inputs play in driving outbreaks.

### Conclusions

In examining the secondary outbreak hypothesis with mtDNA control region markers in the central Pacific, we discovered substantial genetic differentiation in all *A. planci* populations from different regional and archipelagic zones investigated, suggesting that outbreaks in the central Pacific are not triggered by mass dispersal events, as previously proposed [20], but are rather formed from independent events. There is little genetic evidence that outbreaks are composed of a rogue subset of the greater population, thereby suggesting that individuals from a variety of cohorts and populations are mixing to form outbreaks. We could not determine whether outbreaks drive genetic connectivity within archipelagos, but the substantial population structure and general lack of shared haplotypes between archipelagos clearly indicate limited to zero exchange among them. Surprisingly fine-scale structure was found for a species with such a high dispersal potential, suggesting that limited propagule exchange can exist across small spatial scales, regardless of *A. planci* population density, larval production, and the number of available migrants.

The phenomenon of outbreaks occurring at similar times among vastly disjunct areas is probably due to similar climatic, ecological, or anthropogenic conditions, rather than the planktonic dispersal of *A. planci* larvae. Since outbreaks are not spreading among archipelagos, the efficiency and effectiveness of coral reef conservation efforts to control the spread of *A. planci* in the central Pacific can be greatly improved by focusing efforts within archipelagos and islands.

## Supporting Information

Figure S1 **Median-joining haplotype network of Acanthaster planci samples color coded by region.** Corresponding location numbers are in parenthesis. Each circle represents a unique haplotype connected by a line to those that differ by one or more base pairs. Those lines that represent ≥5 bp differences were labeled by barred increments; however, lines are not drawn to scale. Nodes on the lines indicate missing haplotypes. The smallest colored circles represent a singleton haplotype, and the largest circle represents 25 individuals.
(TIF)

Figure S2 **Median-joining haplotype network of Acanthaster planci samples color coded by island with the exception of north central Pacific (NC), which is color coded by the subregions MHI (main Hawaiian Islands) and NWHI (Northwestern Hawaiian Islands).** Corresponding location numbers are in parenthesis. Each circle represents a unique haplotype connected by a line to those that differ by one or more base pairs. Those lines that represent ≥5 bp differences were labeled by barred increments; however, lines are not drawn to scale. Nodes on the lines indicate missing haplotypes. The smallest colored circles represent a singleton haplotype, and the largest circle represents 25 individuals.
(TIF)

Figure S3 **Median-joining haplotype network of Acanthaster planci samples color coded by outbreak and non-outbreak.** Corresponding location numbers are in parenthesis. Each circle represents a unique haplotype connected by a line to those that differ by one or more base pairs. Those lines that represent ≥5 bp differences were labeled by barred increments; however, lines are not drawn to scale. Nodes on the lines indicate missing haplotypes. The smallest colored circles represent a singleton haplotype, and the largest circle represents 25 individuals.
(TIF)

Figure S4 **Median-joining haplotype network of Acanthaster planci samples color coded by coded by habitat at Pearl & Hermes Atoll.** Corresponding location numbers are in parenthesis. Each circle represents a unique haplotype connected by a line to those that differ by one or more base pairs. Those lines that represent ≥5 bp differences were labeled by barred increments; however, lines are not drawn to scale. Nodes on the lines indicate missing haplotypes. The smallest colored circles represent a singleton haplotype, and the largest circle represents 25 individuals.
(TIF)

Table S1 M and θ posterior probability distributions as calculated by Migrate using a Bayesian MCMC simulation.
(DOCX)

Table S2 M and θ posterior probability distributions as calculated by Migrate using a Bayesian MCMC simulation.
(DOCX)
