# Supplementary figures and images for: There's No Place Like Home: Crown-of-Thorns Outbreaks in the Central Pacific Are Regionally Derived and Independent Events
Source: PLoS One. 2012 Feb 17;7(2):e31159. doi: 10.1371/journal.pone.0031159 (PMC3281911; doi:10.1371/journal.pone.0031159)

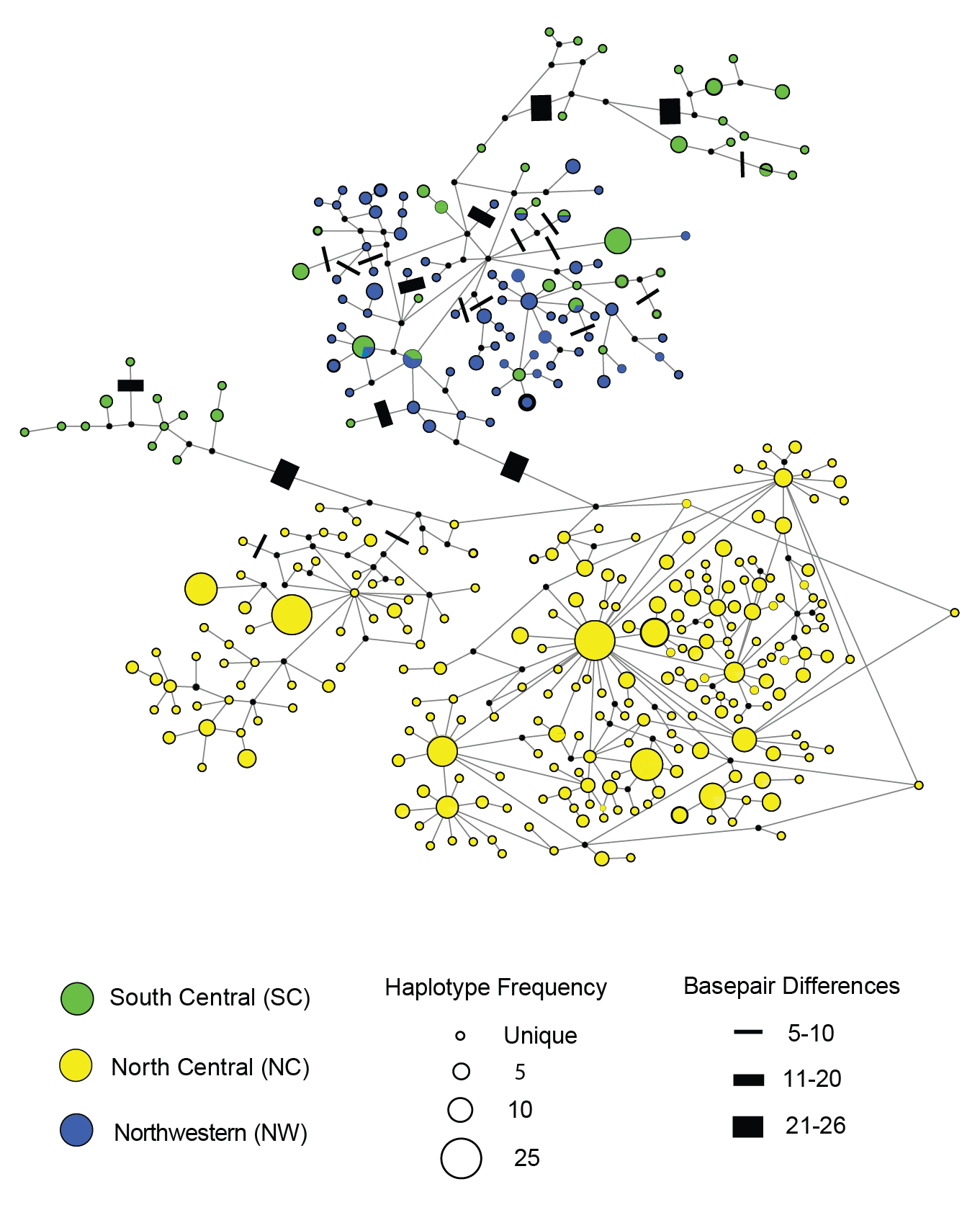

Supplement: Figure S1 — Median-joining haplotype network of Acanthaster planci samples color coded by region. Corresponding location numbers are in parenthesis. Each circle represents a unique haplotype connected by a line to those that differ by one or more base pairs. Those lines that represent ≥5 bp differences were labeled by barred increments; however, lines are not drawn to scale. Nodes on the lines indicate missing haplotypes. The smallest colored circles represent a singleton haplotype, and the largest circle represents 25 individuals. (TIF) [file pone.0031159.s001.tif]

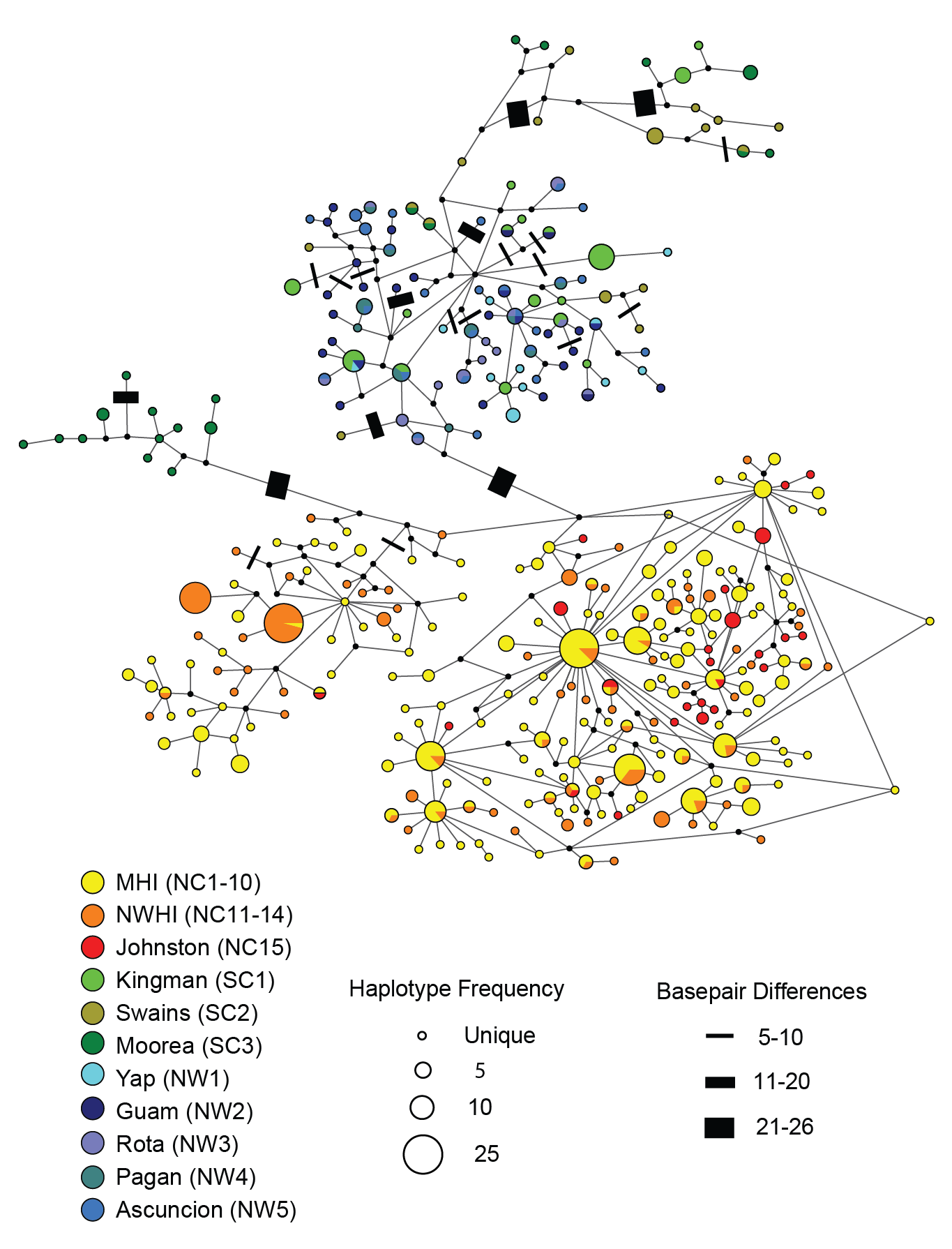

Supplement: Figure S2 — Median-joining haplotype network of Acanthaster planci samples color coded by island with the exception of north central Pacific (NC), which is color coded by the subregions MHI (main Hawaiian Islands) and NWHI (Northwestern Hawaiian Islands). Corresponding location numbers are in parenthesis. Each circle represents a unique haplotype connected by a line to those that differ by one or more base pairs. Those lines that represent ≥5 bp differences were labeled by barred increments; however, lines are not drawn to scale. Nodes on the lines indicate missing haplotypes. The smallest colored circles represent a singleton haplotype, and the largest circle represents 25 individuals. (TIF) [file pone.0031159.s002.tif]

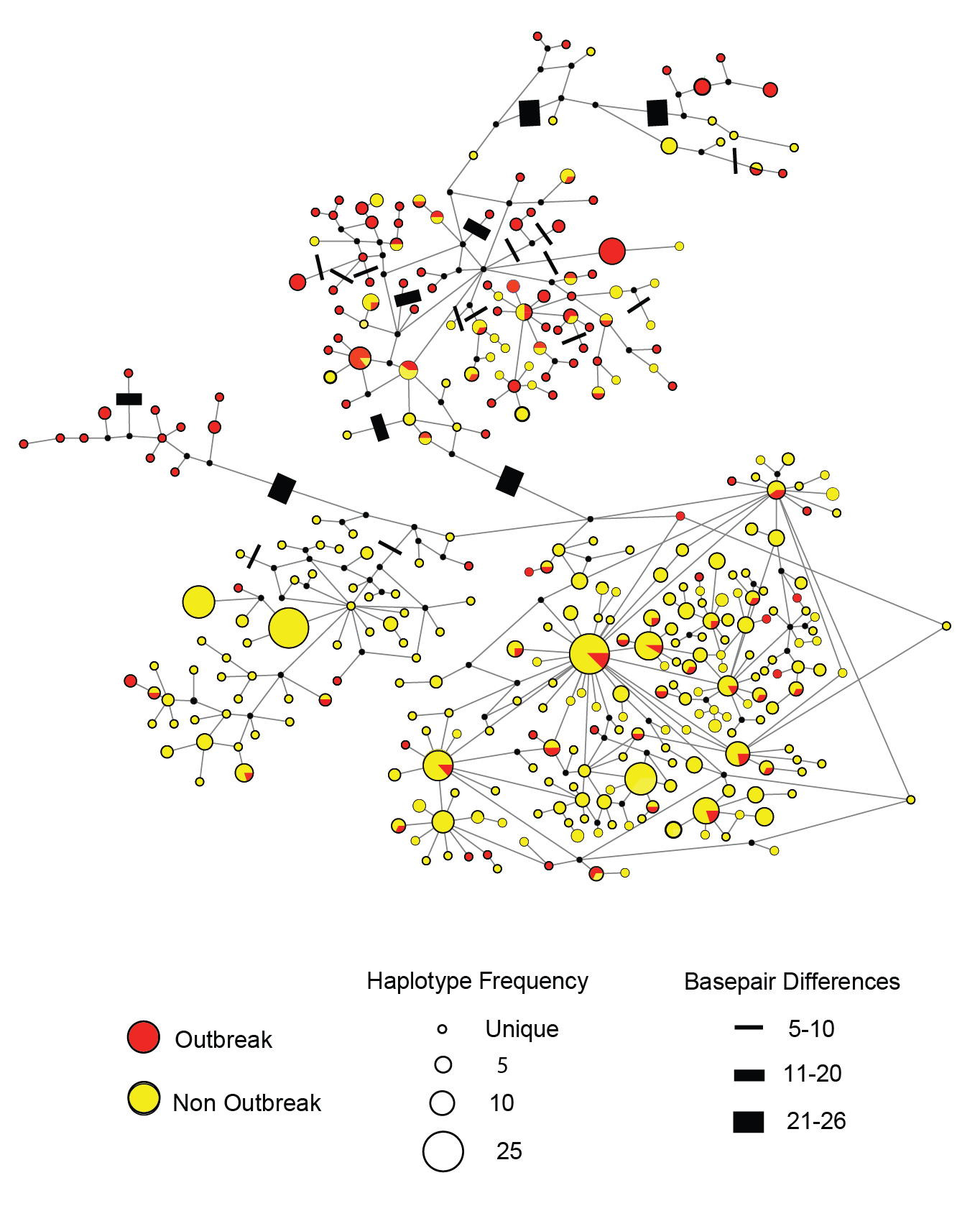

Supplement: Figure S3 — Median-joining haplotype network of Acanthaster planci samples color coded by outbreak and non-outbreak. Corresponding location numbers are in parenthesis. Each circle represents a unique haplotype connected by a line to those that differ by one or more base pairs. Those lines that represent ≥5 bp differences were labeled by barred increments; however, lines are not drawn to scale. Nodes on the lines indicate missing haplotypes. The smallest colored circles represent a singleton haplotype, and the largest circle represents 25 individuals. (TIF) [file pone.0031159.s003.tif]

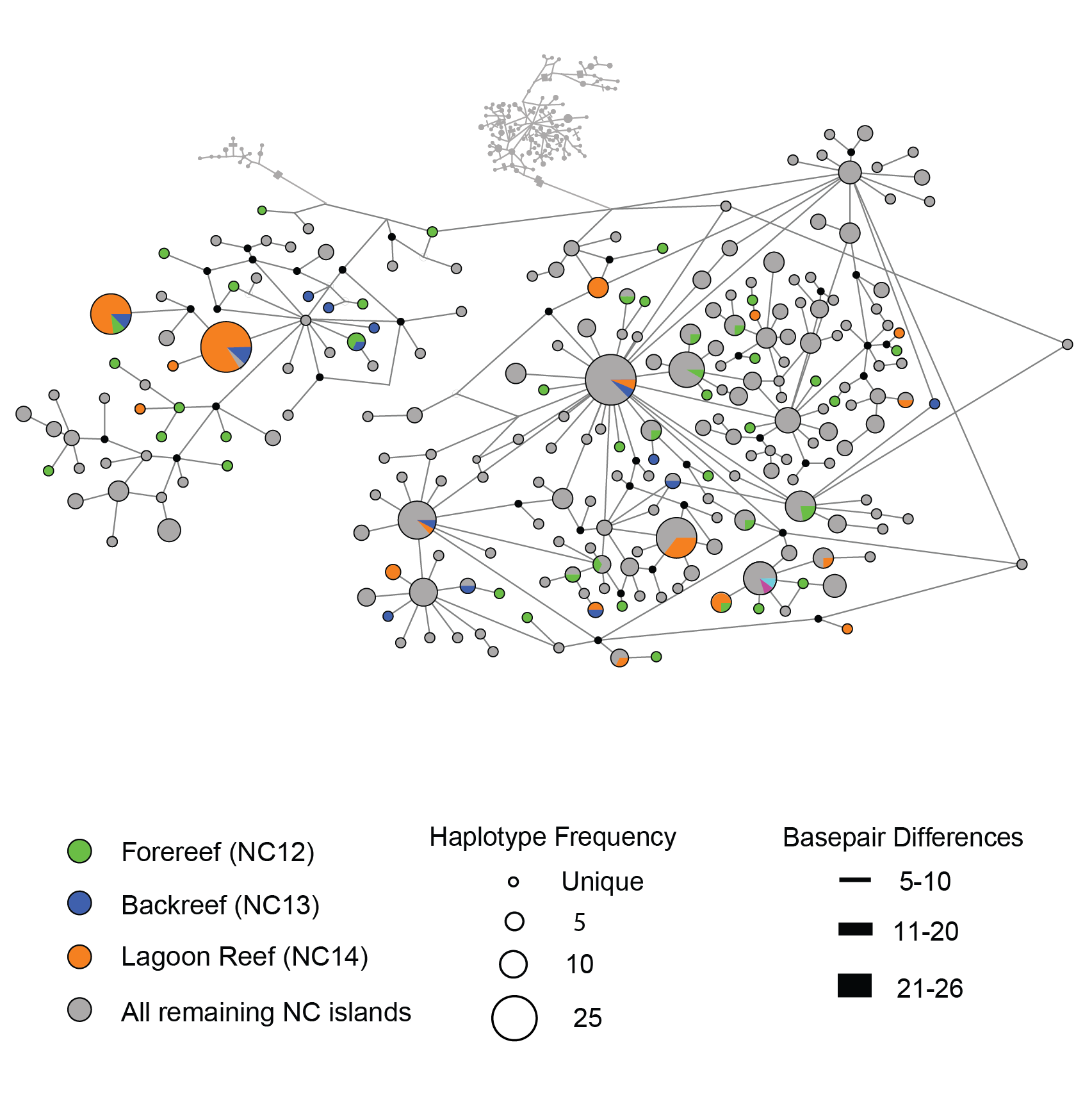

Supplement: Figure S4 — Median-joining haplotype network of Acanthaster planci samples color coded by coded by habitat at Pearl & Hermes Atoll. Corresponding location numbers are in parenthesis. Each circle represents a unique haplotype connected by a line to those that differ by one or more base pairs. Those lines that represent ≥5 bp differences were labeled by barred increments; however, lines are not drawn to scale. Nodes on the lines indicate missing haplotypes. The smallest colored circles represent a singleton haplotype, and the largest circle represents 25 individuals. (TIF) [file pone.0031159.s004.tif]
