# Supplementary material for: There's No Place Like Home: Crown-of-Thorns Outbreaks in the Central Pacific Are Regionally Derived and Independent Events
Source: PLoS One. 2012 Feb 17;7(2):e31159. doi: 10.1371/journal.pone.0031159 (PMC3281911; doi:10.1371/journal.pone.0031159)
Supplement: Table S1 — M and θ posterior probability distributions as calculated by Migrate using a Bayesian MCMC simulation. (DOCX) [file pone.0031159.s005.docx]

**Table S1.** M and θ posterior probability distributions as calculated by Migrate using a Bayesian MCMC simulation.

| **Parameter** | **2.50%** | **25.00%** | **Mode** | **75.00%** | **97.50%** | **Median** | **Mean** |
| --- | --- | --- | --- | --- | --- | --- | --- |
| Q1 | 0.0226 | 0.041 | 0.0527 | 0.0674 | 0.1094 | 0.0589 | 0.06287 |
| Q2 | 0.009 | 0.0226 | 0.0291 | 0.0362 | 0.0512 | 0.0301 | 0.0304 |
| Q3 | 0.0186 | 0.0384 | 0.0527 | 0.0714 | 0.1312 | 0.0621 | 0.06856 |
| M2->1 | 0 | 0 | 0.3 | 30.7 | 122 | 31 | 43.6 |
| M3->1 | 0 | 0 | 0.3 | 58.7 | 471.3 | 59 | 111.5 |
| M1->2 | 0 | 0 | 0.3 | 24 | 200 | 24.3 | 51.8 |
| M3->2 | 0 | 0 | 0.3 | 24 | 303.3 | 24.3 | 67.8 |
| M1->3 | 0 | 0 | 22.3 | 92 | 566.7 | 92.3 | 154.6 |
| M2->3 | 0 | 0 | 0.3 | 54 | 179.3 | 54.3 | 67.6 |

*Population key: 1 = Mo‘orea; 2 = Kingman Reef; 3 = Swains Island
