# Supplementary material for: There's No Place Like Home: Crown-of-Thorns Outbreaks in the Central Pacific Are Regionally Derived and Independent Events
Source: PLoS One. 2012 Feb 17;7(2):e31159. doi: 10.1371/journal.pone.0031159 (PMC3281911; doi:10.1371/journal.pone.0031159)
Supplement: Table S2 — M and θ posterior probability distributions as calculated by Migrate using a Bayesian MCMC simulation. (DOCX) [file pone.0031159.s006.docx]

**Table S2.** M and θ posterior probability distributions as calculated by Migrate using a Bayesian MCMC simulation.

| **Parameter** | **2.50%** | **25.00%** | **Mode** | **75.00%** | **97.50%** | **Median** | **Mean** |
| --- | --- | --- | --- | --- | --- | --- | --- |
| Q1 | 0.17167 | 0.20033 | 0.21292 | 0.22567 | 0.25617 | 0.21325 | 0.21172 |
| Q2 | 0.06583 | 0.09217 | 0.10625 | 0.12117 | 0.15833 | 0.10842 | 0.10874 |
| Q3 | 0.06867 | 0.09017 | 0.10092 | 0.11233 | 0.14 | 0.10242 | 0.10211 |
| M2->1 | 0 | 0 | 0.2 | 4 | 12 | 4.2 | 3.1 |
| M3->1 | 0 | 0 | 0.2 | 4.4 | 14 | 4.6 | 4.2 |
| M1->2 | 0 | 0 | 0.2 | 6.8 | 45.6 | 7 | 12.9 |
| M3->2 | 43.6 | 74.4 | 99 | 134 | 335.6 | 121.8 | 146.9 |
| M1->3 | 0 | 0 | 0.2 | 4.4 | 16.8 | 4.6 | 6.9 |
| M2->3 | 0 | 0 | 8.2 | 20.4 | 242 | 20.6 | 50.3 |

*Population key: 1 = North Central Pacific; 2 = South Central Pacific; 3 = Northwestern Pacific
